# Abietic Acid Enhances the Sedative Activity of Diazepam: In vivo Approach along with Receptor Binding Affinity and Molecular Interaction with the GABAergic System

**DOI:** 10.1002/open.202500397

**Published:** 2025-11-23

**Authors:** Meher Afroz, Rubel Hasan, Mst Muslima Khatun, Mokammel Hossain Tito, Md Shadin, Ranjit Chakma, Mohammed Asiri, Faisal H. Altemani, Abdullah H. Altemani, Md Shimul Bhuia, Muhammad Torequl Islam

**Affiliations:** ^1^ Department of Pharmacy Gopalganj Science and Technology University Gopalganj Bangladesh; ^2^ Bioinformatics and Drug Innovation Laboratory BioLuster Research Center Ltd Gopalganj Bangladesh; ^3^ Department of Medicine Gazipur Agricultural University Gazipur Bangladesh; ^4^ Department of Clinical Laboratory Sciences College of Applied Medical Sciences King Khalid University Abha Saudi Arabia; ^5^ Department of Medical Laboratory Technology Faculty of Applied Medical Sciences University of Tabuk Tabuk Saudi Arabia; ^6^ Department of Family and Community Medicine Faculty of Medicine University of Tabuk Tabuk Saudi Arabia; ^7^ Pharmacy Discipline Khulna University Khulna Bangladesh

**Keywords:** abietic acid, GABAergic system, in vivo, molecular docking, sedation

## Abstract

This study evaluated the sedative activity of abietic acid (AA) through a thiopental sodium (TS)‐induced sleep model in mice. AA (5, 10, and 20 mg/kg) and diazepam (DZP) (2 mg/kg) were provided, followed by TS (20 mg/kg) after 30 min to induce sleep. Sleep latency and total sleeping time were documented over a 4 h period. Additionally, molecular docking studies were conducted to examine the interactions of AA with GABA_A_ (Protein Data Bank: 6X3X) receptors, which hold two subunits of α1 and β2, alongside pharmacokinetic and toxicity assessments. The results indicated that AA significantly (*p *< 0.05) provided the fast onset of sleeping and extended sleeping time in a dose‐dependent manner. The combination of AA (20 mg/kg) with DZP further enhanced sedation, yielding a prolonged sleep duration and a reduced sleep latency, indicating a synergistic effect. In addition, in silico analysis expressed that AA exhibited a strong binding affinity for GABA_A_ receptors (–7.9 kcal/mol), comparable to DZP (–8.4 kcal/mol). Furthermore, AA demonstrated favorable pharmacokinetic properties and drug‐likeness. Overall, these findings suggest that AA possesses potent sedative effects, likely mediated through interactions with the GABAergic system, warranting further investigation for its therapeutic potential in sleep disorders.

## Graphical abstract

Abietic acid (AA) reveals potent sedative potential by expressing fast sleeping onset and increased sleeping duration compare to the control group in thiopental sodium induced sleeping mice. The compound also exhibited notable binding affinity (–7.9 kcal/mol) toward GABAA receptors. In addition, the ligand (AA) showed acceptable pharmacokinetic features and drug‐likeness.

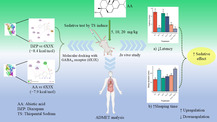

## 1 Introduction

Abietic acid (AA) is a colorless, crystalline organic compound and a primary component of resin found in coniferous trees. It is a natural abietane diterpenoid derived from *Pinus palustris* and *Pimenta racemosa var. grissea*, exhibiting various pharmacological activities, including anti‐inflammatory, anticonvulsant, antiobesity, and antiallergic effects [1, 2–3]. Additionally, AA possesses antibacterial, antiviral, antifungal, neuroprotective (Alzheimer's disease), and antioxidant properties. It also inhibits gastric secretions, suggesting potential as an antiulcer agent [4, 5]. Moreover, AA has demonstrated antiobesity, anti‐inflammatory, antiemetic, anti‐psoriatic, osteoprotective, and neuropathic pain‐relieving effects [6, 7].

Insomnia is a sleep disturbance defined by difficulty in getting to sleep and remaining asleep, or both, despite having the opportunity to do so [8]. Insomnia can be acute (short‐term) and last a few days to weeks, often triggered by stress or life changes, or chronic (long‐term), lasting a month or longer, and may be linked to ongoing stress, underlying medical conditions, or mental health issues [9, 10]. Each month, 30% of people say they experience sleep issues that persist for a few nights or longer [11]. However, in the general population, insomnia can lead to depression in about 60%–80% of depressed patients, and other diseases can involve insomnia, including somatic diseases, sexual problems, type 2 diabetes, hypertension, schizophrenia, and acute myocardial infarction [12, 13]. Some causes of insomnia include the presence of chronic illnesses such as cancer, cardiovascular diseases, stressful life matters, and high depression and anxiety scores [14].

Many neurotransmitters, including gamma‐aminobutyric acid (GABA), serotonin (5‐hydroxytryptamine), histamine, norepinephrine, epinephrine, glutamate, hypocretin, and dopamine, have a significant impact on sleep duration and hence may be an effective therapy for insomnia [15]. GABA is a pivotal neurotransmitter that accelerates sedation and having the main preclusive neurotransmitter in the central nervous system (CNS) [16]. GABA receptors consist of three subtypes: GABA_A_, GABA_B_, and GABA_C_ [17]. Among these subtypes GABA_A_ receptors, which include *α*1, *α*2, *α*3, or *α*5 subunits, function as ligand‐gated ion channels [18]. Through the ligand‐gated ion channel opening, activation of the GABA_A_ receptor causes an increase in the inward migration of chloride ions, which results in hyperpolarization and the inhibition of signal transmission [16, 19]. When potassium ions flow into the presynaptic cell, the membrane becomes hyperpolarized. This reduces the entry of calcium ions (Ca^2+^) and, as a result, less neurotransmitter is released [20].

Sedative and anxiolytic medications, including benzodiazepines (BZDs) and barbiturates, exert their effects via GABA on the GABA_A_ receptor [21, 22]. BZD has been extensively used in the management of several conditions, including anxiety, muscular relaxation, insomnia, seizure disorders, and as general anaesthetics [23]. BZD interacts with binding sites located between the *α* and *γ* subunits of the GABA_A_ receptor to enhance GABAergic transmission. While BZD effectively promotes and sustains sleep, prolonged usage results in several adverse effects, including headache, impaired coordination, confusion, insomnia, weakness, disorientation, and drowsiness [24]. Additionally, barbiturates are a class of sedatives often used to manage insomnia by modulating CNS activity and stimulating the *α* and *β* subunits of the GABA_A_ receptor. Barbiturates may augment chloride ion influx and activate GABA_A_ receptors at minimal doses of GABA. Prolonged use of barbiturates may produce detrimental side effects, such as agitation, hallucinations, disorientation, headaches, and drowsiness [25, 26]. However, this research aimed to assess the sedative activity of AA on Swiss albino mice. In addition, we also performed an in silico study to determine the possible molecular interactions behind the observed effect.

## 2 Materials and Methods

### 2.1 In vivo Study

#### 2.1.1 Chemicals and Reagents

Water for injection (WFI), diazepam (DZP), and thiopental sodium (TS) were purchased from Opsonin, Square, and ACI Pharmaceuticals Ltd., Bangladesh, respectively. Both AA (CAS No. 514‐10−3) and Tween 80 (Catalogue No. 822 187) were collected from Sigma–Aldrich (USA) and Chengdu Alfa Biotechnology Co. Ltd. (China), respectively.

#### 2.1.2 Section and Preparation of Test and Control Groups

Based on our toxicity analysis, we selected three (lower, middle, and higher) doses of the test compound (AA). Initially, we developed a mother solution with a greater dose of this drug at a dose of 20 mg/kg [27], then diluted it to make intermediate and lower doses (10 and 5 mg/kg) by adjusting with WFI. The WFI and DZP solutions were properly mixed to formulate a dose of 2 mg/kg, serving as the reference preparation. In combination therapy, a higher dose of AA and DZP was administered for co‐treatment to elucidate their synergistic impact.

#### 2.1.3 Experimental Animals

*Swiss* albino mice (21–25 g) were purchased from the animal centre at Jahangirnagar University, Savar, Bangladesh. Prior to the conduct of the study, the animals were maintained at a fixed temperature of 28 ± 1°C (relative humidity: 65%) with regulated lighting (12 h dark/light cycle) in the pharmacology laboratory of the pharmacy department at Gopalganj Science and Technology University, Gopalganj 8100, Bangladesh. The animals always had free access to reliable food and water sources. The present investigation was carried out from 7:30 a.m. to 2:30 p.m., and the mice were inspected for 17 h to observe any potential poststudy fatalities. The Animal Ethics Committee at Gopalganj Science and Technology University, Gopalganj 8100 (GSTU) (#Gstu/phrt17−02).

#### 2.1.4 In Vivo Protocol

Thirty animals in all were randomly split into six groups, each consisting of five animals, following a 4‐day adjustment period (see Table 1). Intraperitoneal (i.p.) administration of the vehicle (control), standard medication (DZP), and test sample was performed. Each animal was placed in an observation room, such as a plastic cage, after receiving an intraperitoneal injection of 20 mg/kg body weight of TS to induce sleep after a 30 min treatment time. The righting reflex was eliminated, and the latency was recorded subsequent to the TS injection. The length of an individual's sleep or the period between awakening and restoration of responsiveness was carefully documented by manual observation. The treatments and dosages are enumerated in Table 1.

**TABLE 1:** Various test groups and their doses.

Treatment groups	Dose (mg/kg) (p.o)	Target receptor
NC (Vehicle)	10	—
DZP	2	GABA
AA	5	Under Investigation
	10	
	20	
AA + DZP	20 + 2	–

AA: Abietic acid; DZP: Diazepam; NC: Negative control; and GABA: Gama amino butyric acid.

#### 2.1.5 Statistical Analysis

All statistical analyses were performed using Python (version 3.11; libraries: pandas 2.2.2, seaborn 0.13.2, matplotlib 3.9.2) and R (version 4.4.1; packages: ggplot2 3.5.1, ggpubr 0.6.0, dplyr 1.1.4, multcomp 1.4–25, and ggsci 3.2.0). Data are expressed as the mean ± standard error of the mean (SEM). Statistical significance was assessed using one‐way analysis of variance (ANOVA), followed by Tukey's post hoc test for multiple comparisons. For statistical significance, a threshold of *p* < 0.05 was applied. Data visualization (violin plots, bar graphs with error bars, and heatmaps) was conducted using the above‐mentioned R and Python libraries to illustrate group distributions, variability, and mean differences.

### 2.2 In Silico Analysis

#### 2.2.1 Selection and Preparation of GABA_A_ Macromolecule

According to earlier research, we selected the two subunits (*α*1 and *β*2) of the GABA_A_ receptor, which mediate sedation (Brohan and Goudra, 2017). The Protein Data Bank (PDB) is the only global organisation that collected and made available the 3D configurations of macromolecules in biological systems and their complexes. The online application serves as a sophisticated resource for investigating biological disciplines [28]. The 3D configuration of GABA_A_ receptor subunits (*β*2 and *α*1 in the A and B chain, respectively) was acquired from the PDB (PDB ID: 6X3X) on June 23, 2024. In order to minimize docking interference after collection, the receptors have been optimized. By removing unnecessary amino acid residues, water molecules, and heteroatoms, PyMOL version 1.7.4.5 was utilised to improve the macromolecules [29]. Following that, the Swiss‐PDB Viewer online tool was used to build the protein structure by modifying the GROMOS96 43 B1 force field [30, 31].

#### 2.2.2 Collection and Preparation of Ligands

**FIGURE 1:**
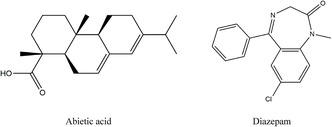
Chemical structure of AA and DZP.

Both ligand (AA and DZP) structures were gathered from the online server PubChem (https://pubchem.ncbi.nlm.nih.gov/) with their PubChem ID: 3016, respectively, and saved in ‘sdf’ file format. Then, the ligands were prepared by minimizing energy with Chem3D Pro21.0 software [32]. The 2D conformation of DZP and AA is presented in Figure 1.

#### 2.2.3 Docking Protocol and Non–bond Interaction

The molecular docking approach is a usual computational tool used today in pharmaceutical research for drug development [33]. This method assesses the pharmacodynamic characteristics of drugs by analyzing and correlating molecules to certain binding sites utilizing the PyRx v0.8 computer program [30, 34]. The results of the docking method show how strongly a ligand interacts with a specific protein's active region. To accelerate the docking procedure, the X, Y, and *Z* axes of the grid box were resized to 35.02 × 39.37 × 25.00 Å, respectively [31]. Afterward, the calculation was performed 2000 times. The ligand in PDBQT format was obtained by acquiring the PDB format of the ligand–protein complex [35]. The binding affinity value of the ligand was given as a negative value in kcal/mol. Additionally, BIOVIA Discovery Studio v21.1.0 is used to analyze the proteins’ active binding regions. This software enables the assessment of nonbond interactions in ligand‐protein complexes [27, 29].

#### 2.2.4 Prediction of Pharmacokinetics and Drug‐Likeness

Pharmacokinetics, which refers to the way a drug molecule is processed in the body, is often analyzed by examining several features that influence its capacity. The absorption, distribution, metabolism and elimination (ADME) characteristics,estimated early on during the discovery period, significantly decrease the occurrence of pharmacokinetics‐related failures in the subsequent clinical phases [36, 37]. To estimate the adbsorption, distribution, metabolism, elimination and toxicity (ADMET) characteristics and drug‐likeness parameters, the SwissADME online server can be used [6].

#### 2.2.5 Toxicity Prediction

Toxicity prediction is a crucial stage in the drug discovery approach that aids in the prioritization and identification of chemicals with the most potential for efficient and safe usage in humans. This technique also helps minimize the likelihood of expensive failures in the later stages of drug discovery [38]. Any given drug's toxicity features can be predicted using the ProTox 3.0 online tool. The PubChem canonical SMILES of every compound were then submitted to the ProTox 3.0 website (http://tox.charite.de/protox‐3) for evaluation of the chemical toxicity. The website then identified the compound's toxicity category and a number of other characteristics. Table 2 presents the analysis and documentation of the selected chemical's various toxicity parameters.

**TABLE 2:** The toxicological profiles of DZP and AA by ProTox 3.0.

Parameters	Diazepam	Abietic acid
Predicted toxicity Class	2	4
Hepatotoxicity	Inactive	Active
Nephrotoxicity	Inactive	Inactive
Respiratory toxicity	Active	Active
Cardiotoxicity	Inactive	Inactive
Immunotoxicity	Inactive	Inactive
Neurotoxicity	Active	Active
Carcinogenicity	Inactive	Inactive
Mutagenicity	Inactive	Inactive
Nutritional toxicity	Inactive	Inactive
Ecotoxicity	Active	Active
Cytotoxicity	Active	Inactive
Blood brain barrier	Active	Active
Clinical toxicity	Active	Inactive
Predicted LD_50_	48 mg/kg	1000 mg/kg

## 3 Results

### 3.1 In vivo Investigation

**FIGURE 2:**
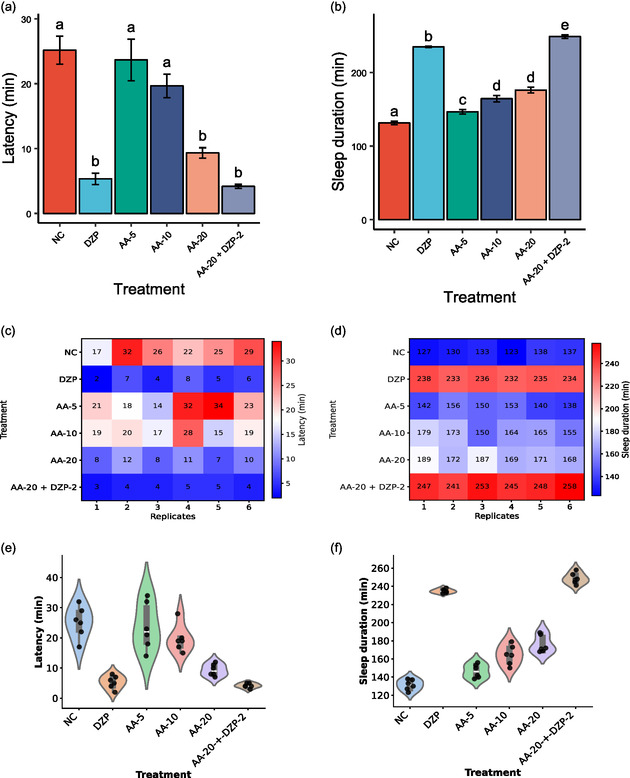
One‐way ANOVA followed by Tukey's HSD as a post hoc test with multiple comparisons among treatment groups of the latency period and duration of sleeping of the sedative activity (values are mean ± SEM, *n* = 5). (a) Groups that share at least one letter are not statistically significantly different (*p *< 0.05) in the latency period of the treatment group. (b) Groups that share at least one letter are not statistically significantly different (*p* < 0.001) in the sleeping duration (min), except between AA‐5 and AA‐10 (*p *< 0.01), DZP + AA‐20 − DZP‐2 (*p *< 0.05), and NC − AA‐5 (*p *< 0.05). (c,d) Heatmap of both latency and sleep duration. (e,f) Biolin plot of both latency and sleep duration.

According to the in vivo study, the vehicle (NC) group exhibited a prolonged latency period (25.17 ± 2.15 min), whereas the reference group (DZP) significantly reduced (*p *< 0.05) the latency period to 5.33 ± 0.88 min compared to the NC group. Treatment with AA produced a significant (*p *< 0.05) dose‐dependent reduction in latency, with the values of 23.67 ± 3.21, 19.67 ± 1.81, and 9.33 ± 0.80 min for AA‐5, AA‐10, and AA‐20 mg/kg, respectively. Although AA showed its efficacy lower than DZP at equivalent doses but it had a clear sedative effect. Furthermore, the combination of AA‐20 + DZP resulted in the shortest latency (4.17 ± 0.31 min), surpassing the effect of DZP alone, indicating a potential synergistic interaction between AA and DZP. The latency periods for all test and control groups are presented in Figure 2a.

In addition to reducing sleep latency, AA significantly prolonged sleep duration in a dose‐dependent manner. The vehicle (NC) group exhibited a mean sleep duration of 131.33 ± 2.37 min, while DZP significantly (*p *< 0.05) increased this duration to 234.67 ± 0.88 min. Treatment with AA resulted in sleep durations of 146.50 ± 3.05, 164.33 ± 4.04, and 176.00 ± 3.84 min for AA‐5, AA‐10, and AA‐20 mg/kg, respectively. Although the sleep‐promoting effect of AA was lower than that of DZP, the progressive increase across doses indicates a dose‐dependent sedative action. Interestingly, the combined treatment (AA‐20 + DZP) produced the longest sleep duration (248.66 ± 2.45 min), exceeding that of DZP alone. The sleep durations for all experimental groups are illustrated in Figure 2b.

**FIGURE 3:**
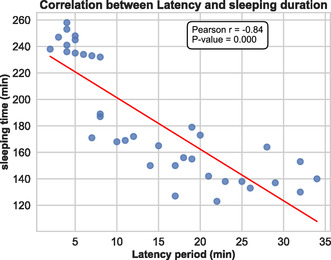
Correlation between latency and sleep duration across sedative treatments via scatter plot with trend line.

Figure 3 presents a scatter plot illustrating a negative correlation between sleep latency (in minutes) and sleep duration. As latency increases, sleep duration decreases. The Pearson correlation coefficient (*r* = −0.84) indicates a strong negative relationship, and the *p*‐value (*p* = 0.000) confirms that this correlation is statistically significant. The red trend line visually emphasizes this inverse association.

### 3.2 In Silico Study

#### 3.2.1 Molecular Docking

**FIGURE 4:**
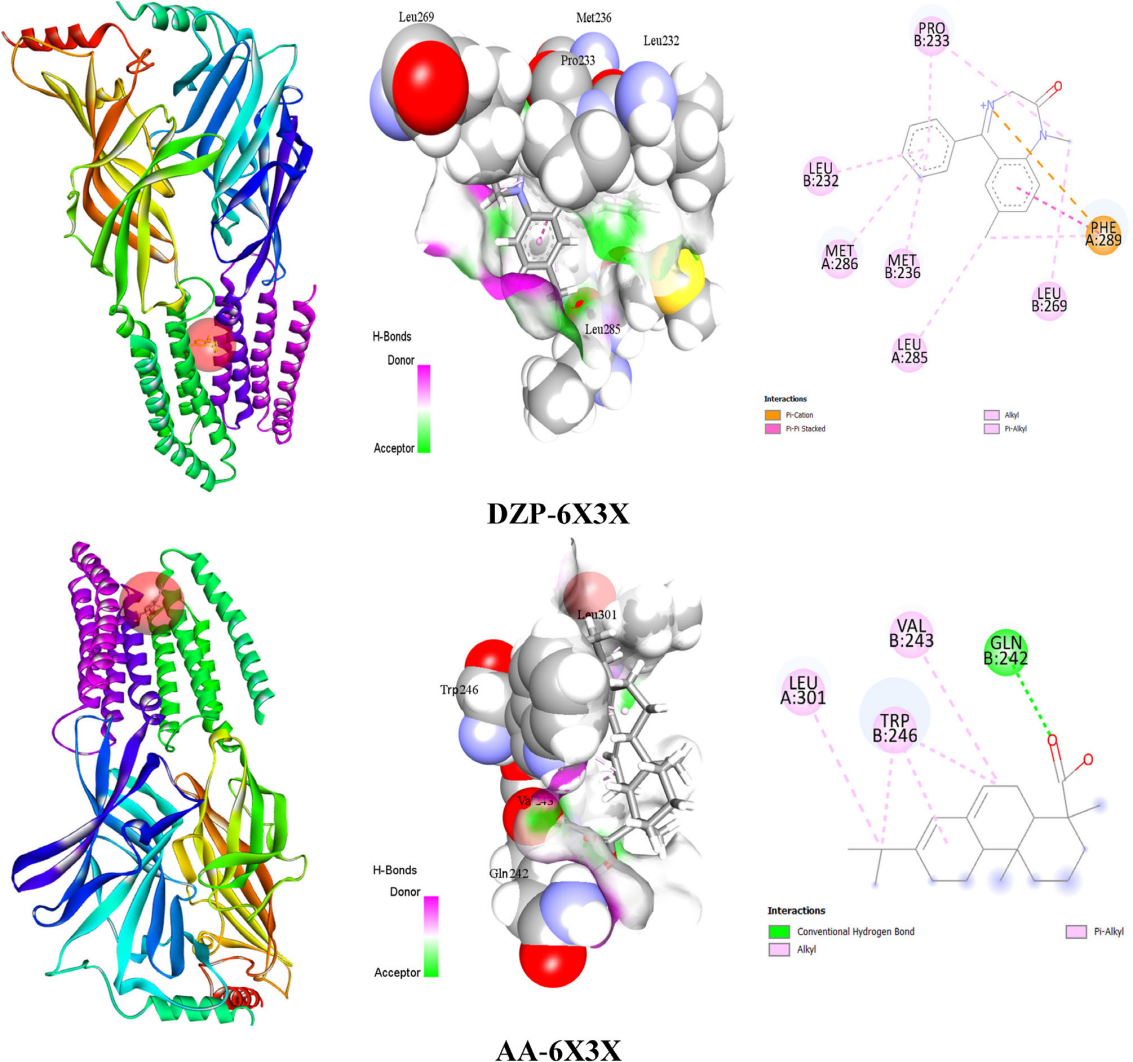
3D and 2D visualization of DZP and AA interaction with the GABA_A_ receptor. [AA: Abietic acid; DZP: Diazepam].

According to the in silico study, the conventional drug DZP binds with the same receptor with a docking score of –8.4 kcal/mol, but the tested ligand (AA) had a binding score of –7.9 kcal/mol against the GABA_A_ receptor. The results also showed that the tested ligand (AA) bound to the GABA_A_ receptor by forming a single hydrogen bond (HB) with GLN242, along with HPs with amino acid residues of LEU301 (Alkyl), VAL243 (Alkyl), and TRP246 (Pi‐Alkyl). Although DZP didn’t form any HB, several hydrophobic (HP) bonds formed with the presence of amino acid residues of PHE289 (Pi–Pi Stacked), MET236 (Alkyl), PRO233 (Alkyl), MET286 (Pi‐Alkyl), LEU232 (Pi‐Alkyl), MET236 (Pi‐Alkyl), LEU285 (Alkyl), PRO233 (Pi‐Alkyl), and PHE289 (Pi‐Alkyl). The binding scores, bond types, number of hydrogen bonds, hydrogen bond lengths, and a list of amino acid residues involved in ligand–receptor interactions are illustrated in Table 3. Figure 4 shows both the binding sites and 2D and 3D views.

**TABLE 3:** Binding value of AA and DZP toward GABA_A_ (PDB) receptors.

Ligands	**Docking value (kcal/mol) with GABA**_**A**_ **(6X3X)**	No. of HB	HBs residues	HB length (Å)	Other bond residues
DZP	−8.4	**–**	**–**	**–**	PHE289 (Pi–Pi Stacked), MET236 (Alkyl), PRO233 (Alkyl), LEU285 (Alkyl), LEU232 (Pi‐Alkyl), MET236 (Pi‐Alkyl), MET286 (Pi‐Alkyl), PRO233 (Pi‐Alkyl), PHE289 (Pi‐Alkyl)
AA	–7.9	1	GLN242	2.25	LEU301 (Alkyl), VAL243 (Alkyl), TRP246 (Pi‐Alkyl)


AA: Abietic acid; DZP: Diazepam; HB: Hydrogen bond; GABA: Gama amino butyric acid.

#### 3.2.2 Estimation of in Silico Pharmacokinetics and Drug‐Likeness

**FIGURE 5:**
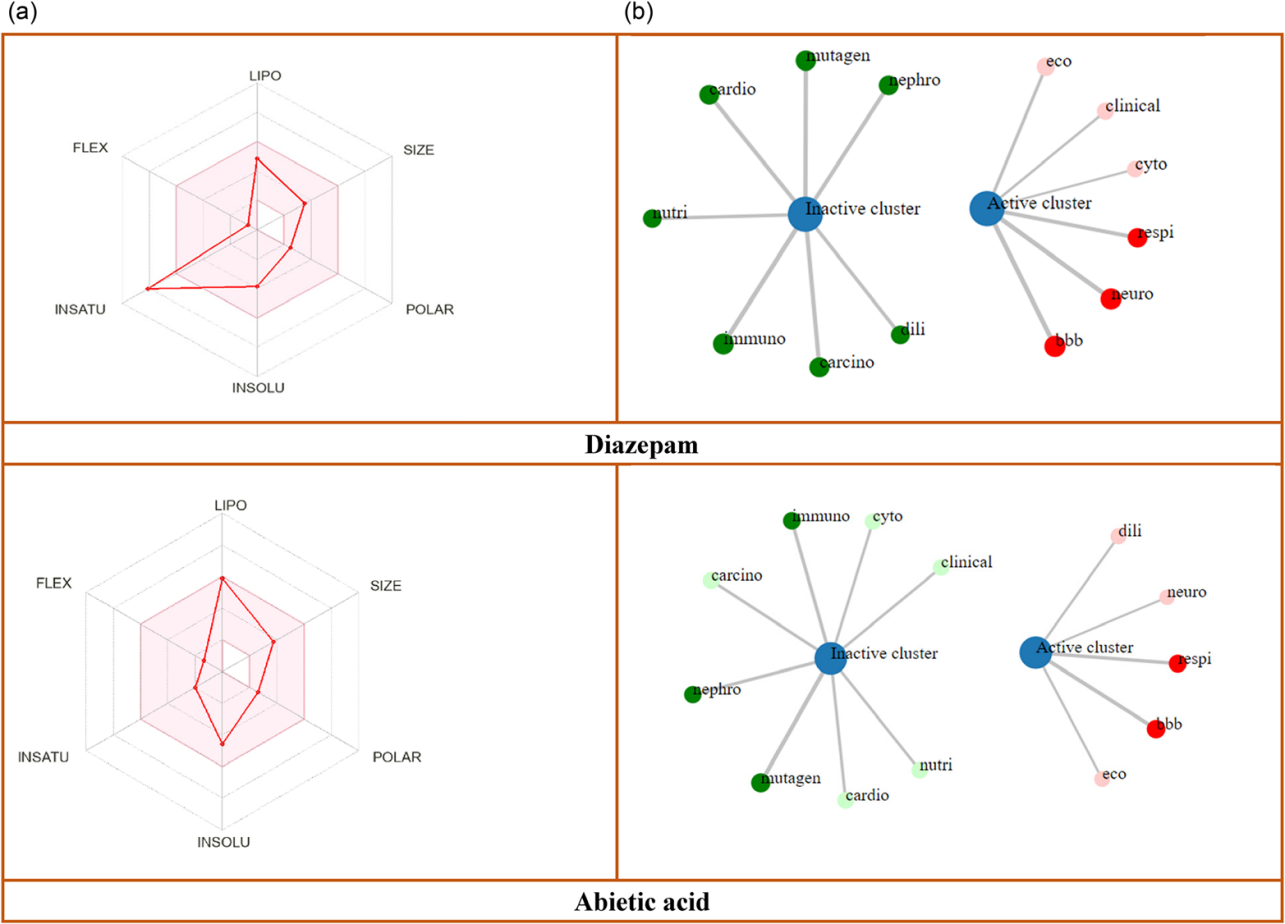
(a) An overview of the pharmacokinetic, toxicological, and physiochemical characteristics of AA and DZP [for oral bioavailability, the coloured zone is the ideal physicochemical region]; SIZE: 150 g/mol < MV < 500 g/mol; INSOLU (Insolubility): −6 < log S (ESOL) < 0; POLAR (Polarity): 20 Å^2^ < TPSA < 130 Å^2^; LIPO (Lipophilicity): −7 < XLOGP3 < +5.0; FLEX (Flexibility): 0 < num. rotatable bonds < 9] INSATU (In saturation): 0.25 < Fraction Csp3 < 1. (b) The purpose of the network chart is to graphically depict the connections between the chosen substances, DZP and AA, and their anticipated biological functions. [cardio: cardiotoxicity; nutri: nutritional toxicity; nephro; nephrotoxicity; mutagen: mutagenicity; carcino: carcinogenicity; dili: drug induced liver injury; neuro: neurotoxicity; immuno: immunotoxicity; bbb: blood–brain barrier; clinical: clinical toxicity; eco: ecotoxicity; respi: respiratory toxicity; and cyto: cytotoxicity].

The SwissADME and pkCSM web servers were used to estimate the pharmacokinetics of AA and DZP in silico. Both substances met the requirements to be considered as possible medications since they showed promising drug‐likeness characteristics. Molecular weights, 302.45 and 284.74 g/mol for AA and DZP, respectively, were within the acceptable range of less than 500 g/mol. In addition, they complied with Lipinski's rule of five, indicating optimal physicochemical properties with no violations, as shown in Table 4. Additionally, the investigation indicated that AA dissolves in water more readily than DZP. Additionally, both DZP and AA exhibited high absorption in the gastrointestinal tract, supporting their potential for oral bioavailability. Additional pharmacokinetic parameters such as P‐glycoprotein (P‐gp) substrate classification, blood–brain barrier (BBB) permeability, and CYP2C19 inhibition are summarized in Table 4, with corresponding graphical representations shown in Figure 5.

**TABLE 4:** Various pharmacokinetic parameters of AA and DZP were estimated by the SwissADME and pkCSM web servers.

Parameters	Diazepam	Abietic acid
MF	C_16_H_13_ClN_2_O	C_20_H_30_O_2_
MW	284.74 g/mol	302.45 g/mol
LogP	2.67	4.54
HBA	2, 3	2
HBD	0	1
MR	87.95	92.22
GI absorption	High	High
Water solubility	−4.196	−3.939
BBB permeability	0.331	0.353
CNS permeability	−1.397	−2.029
Lipinski	Yes; 0 violation	Yes; 1 violation
Synthetic accessibility	3.00	4.80
P‐gp substrate	No	No
Bio Score	0.55	0.85
CYP2C19 int	Yes	Yes

MW = Molecular weight (g/mol) (optimum = ≤500); MF = Molecular formula; HBA = Hydrogen bond acceptor (optimum = ≤10); LogP = Log P*o/w* (MLOGP) (optimum = ≤5); HBD = Hydrogen bond donor (optimum = ≤5); MR = Molar refractivity (optimum = ≤140); BIO Score = Bioavailability Score; CYP2C19 int = CYP2C19 inhibitor.

#### 3.2.3 Assessment of in Silico Toxicity of the Selected Compound

In the toxicological analysis, the reference ligand, DZP, was classified as toxicity class 2, whereas the tested compound AA was placed in toxicity class 4. The LD_50_ value for DZP was 48 mg/kg, while AA demonstrated a considerably higher LD_50_ of 1000 mg/kg, suggesting that AA has a more favorable safety profile. Both compounds revealed the positive result for neurotoxicity, respiratory toxicity, BBB permeability, and ecotoxicity. However, neither compound demonstrated toxicity concerns, as both were predicted to be inactive for nephrotoxicity, immunotoxicity, cardiotoxicity, carcinogenicity, mutagenicity, and nutritional toxicity. The toxicological properties for DZP and AA are summarized in Table 2, and their interaction network is illustrated in Figure 5.

## 4 Discussion

The GABAergic system functions as the principal inhibitory manner in the brain, which is important for multiple neurological functions, including neurogenesis, neuroapoptosis, and neuronal development (Wu and Sun, 2015). Abnormalities in the GABAergic system may significantly influence the pathophysiology of several mental disorders, such as depression [39]. However, TS is a medicinal agent that has neuroprotective effects on the brain. It serves as a preanaesthetic in surgical procedures for the management of many medical disorders, including insomnia and seizures [12, 40]. Due to the mentioned characteristics, TS‐induced sleep studies may be extensively used to evaluate sedative, hypnotic, or antidepressant effects. The animal model uses nonhuman animals to replicate disease development, diagnosis, and treatment analogous to people [32, 41]. The efficacy of animal models in pharmacological research was shown via the assessment of murine models. The current research used both male and female Swiss albino mice [42]. Current research indicates that DZP exhibits sedative properties via its action on the GABA_A_ receptor [43]. It has been shown to enhance the activation of GABA_A_ receptors [44], resulting in the influx of Cl− into the cell. The sedative effect may be shown at a dosage of 2 mg/kg (i.p.) in animals [45, 46–47]. Therefore, chemicals that reduce the sleep onset and prolong the period of sleep‐in animals treated with DZP are sedative medicines that exert their actions by interacting with GABA_A_ receptors [22, 44, 48].

The provided in vivo data suggest that the experimental compound (AA) exhibits a dose‐dependent sedative action, similar to the reference drug (DZP), as it significantly reduced sleep onset latency and increased sleep duration in a dose‐dependent manner. Among all the doses, the higher dose (AA‐20 mg/kg) showed the strongest sedative effect, with a short latency (9.33 ± 0.80 min) and the longest sleep duration (176 ± 3.84 min) compared to the lower doses of AA and the negative control (NC) group. Although AA produced a clear dose‐dependent sedative effect, its potency was lower than DZP, suggesting that AA may have limited clinical relevance as a standalone sedative. However, its ability to enhance TS‐induced sedation indicates potential as an adjunct or potentiator rather than a primary therapeutic agent.

Synergistic effects are produced when two or more components (such as drugs, chemicals, or biological variables) combine to create an effect that is larger than the sum of their separate effects [49]. These findings suggest a synergistic interaction between AA and DZP, as the combination (AA‐20 + DZP) produced a shorter latency (4.17 ± 0.31 min) and longer sleep duration (248.66 ± 2.45 min) than DZP alone, indicating potentiation of sedative activity.

**FIGURE 6:**
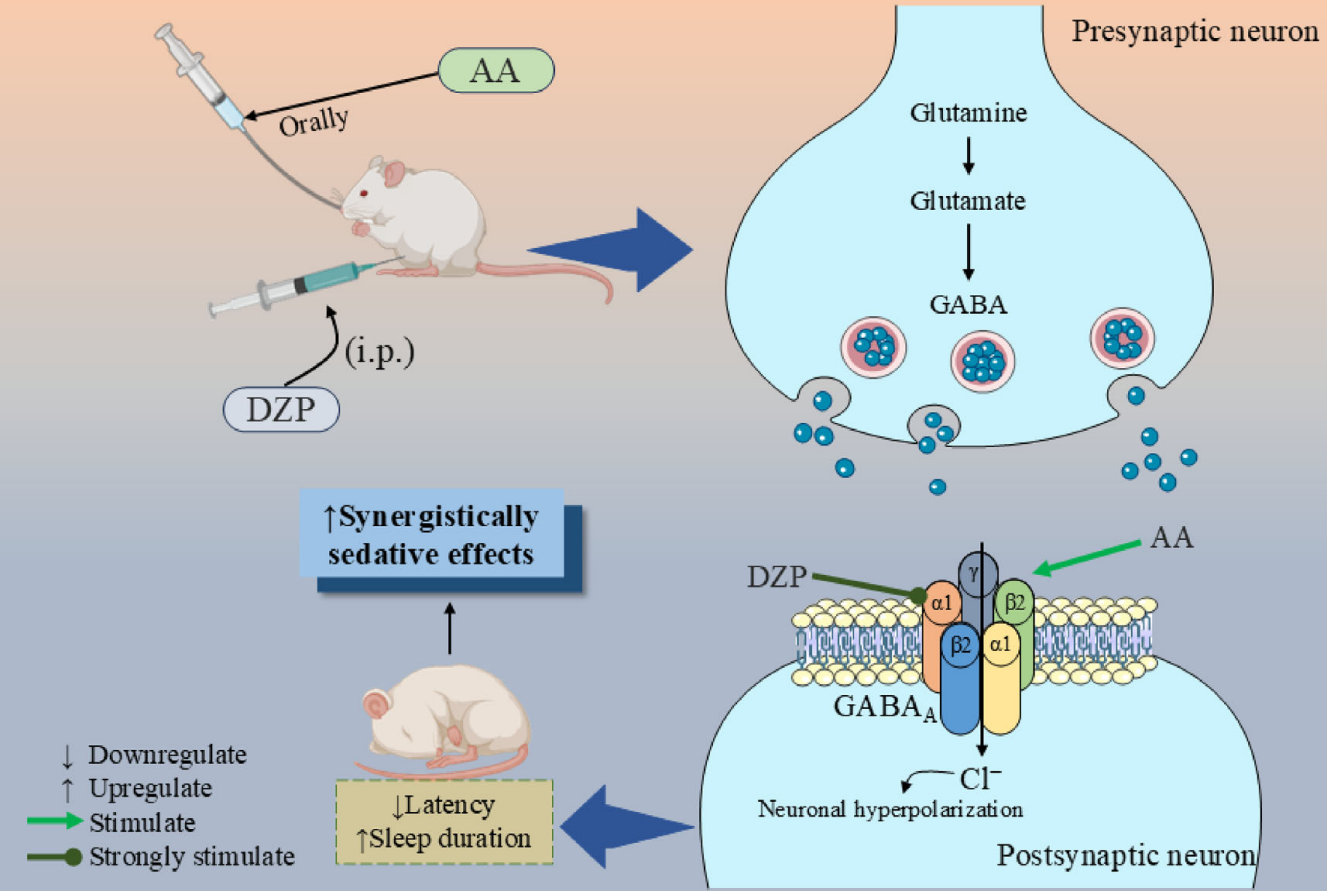
Potential sedative mechanism of AA and DZP with GABAergic pathways. [AA: Abietic acid; DZP: Diazepam; and GABA: Gama amino butyric acid].

Molecular docking is a computer method utilized for predicting the compatibility of two or more molecular structures, such as a medication and a protein, facilitating the comprehension and prediction of molecular interactions and the development of medicinal treatments. Additionally, it saves time and money by identifying potential therapeutic candidates and explaining how medications work [33, 50]. The in silico analysis demonstrated that the standard ligand (DZP) bound with the GABA_A_ receptor by expressing the binding value of –8.4 kcal/mol and also developed different types of HP bonds that were involved with specific amino acid residues of PHE289 (Pi–Pi Stacked), LEU285 (Alkyl), PRO233 (Alkyl), MET236 (Alkyl), MET286 (Pi‐Alkyl), LEU232 (Pi‐Alkyl), MET236 (Pi‐Alkyl), PRO233 (Pi‐Alkyl), and PHE289 (Pi‐Alkyl). On the other hand, our selected ligand demonstrated the docking value of –7.9 kcal/mol, which is approximately the same as the reference ligand. It is also noticeable that AA formed one HB bond (GLN242) with the bond distance of 2.25 Å, while DZP didn’t form any HB bond. In addition, AA also developed three HP bonds with the specific residues of LEU301 (alkyl), VAL243 (alkyl), and TRP246 (pi‐alkyl). HB interactions are essential for determining the selectivity of ligand binding and are the primary factor in the development of a stable complex between biomacromolecules and their target ligands [51]. Moreover, hydrogen bonding substantially improves ligand binding and enhances the therapeutic significance of these ligands [52]. However, the bond is classified as a strong interaction if the distance spans between 2.5 and 3.1 Å. On the other hand, a bond is deemed weak if the distance lies between 3.1 and 3.55 Å. [53] According to the *in silico* study, substantial interactions were suggested by the hydrogen bonds that were created, which ranged between 2.5 and 3.1 Å. Figure 6 depicts the potential pathways of AA and DZP.

For drug development to be effective, it is essential to identify molecules having drug‐like qualities since this helps eliminate compounds with poor pharmacokinetic properties or possible toxicity concerns early on. [54, 55] Early detection of substances with weak drug‐like qualities helps researchers avoid expending money on substances that are unlikely to be successful in clinical trials. [56] The pharmacokinetics study revealed that the selected compound (AA) has the higher GI absorption property. The compound, which has higher GI absorption, can provide efficient oral therapy with high bioavailability, predictable dosing, faster therapeutic effects, and improved patient compliance. [57] Lipinski's Rule of Five is a widely used guideline for predicting the pharmacokinetic properties and drug‐likeness of chemical compounds, particularly their oral bioavailability. These parameters help in assessing whether a compound has suitable properties for absorption and permeation. While not absolute, violations of more than one of these rules often indicate potential problems with bioavailability. Furthermore, the molar refractivity (MR) of AA and DZP, two ligands, is 92.22 and 87.95, respectively, falling within the range (MR ≤ 140). By Lipinski's criterion, all ligands are expected to possess exceptional pharmacokinetic properties and come within the range of potentially becoming medicines. The selected chemical exhibits improved pharmacokinetic characteristics and satisfies every requirement outlined by Lipinski's rule of five. Furthermore, the CNS‐active drugs should be maintaining the optimum BBB permeability (logBB) and CNS penetration (logPS) scores (Rankovic et al., 2015); the normal range of logBB values ≥ 0.3 can readily penetrate the BBB, and the logPS value is –2.0 to –1.0 [58, 59]. The pharmacokinetic analysis demonstrated that the selected compound (AA) displayed a logBB score of 0.353 and a logPS score of −2.029, respectively. According to the ADMET interpretation, the AA showed these two parameters’ values within the optimum range; it means the AA can be considered as a sedative drug.

To evaluate the possible harmful effects of chemicals, toxicology testing is crucial. For example, long‐time exposure to chemicals in humans often results in negative consequences such as immune system impairment, genetic material damage, cancer‐causing qualities, and detrimental effects on development and reproduction [60, 61–62]. According to this toxicological analysis, AA didn’t show any harmful impacts in terms of hepatotoxicity, nephrotoxicity, cardiotoxicity, carcinogenicity, immunotoxicity, and mutagenicity. Additionally, the predicted LD_50_ value of AA was 1000 mg/kg, while DZP had 48 mg/kg. A chemical's immediate or acute toxicity in the strain, sex, and age group of a certain animal species under test is measured by the LD_50._ [63] The lower the LD_50_ value, the more toxic the compound, as it takes a smaller amount to be lethal. Conversely, a higher LD_50_ value indicates lower toxicity, as a larger dose is required to produce the same lethal effect [64, 65]. However, it is clear that the test ligand (AA) has a higher lethal dose (LD_50_) than DZP when the two substances are given in the same proportions, indicating that it is less hazardous.

Despite attempts to standardize circumstances, a major limitation is the possible impact of uncontrollable elements like light, sound, or the presence of observers. Additionally, we conducted the in vivo experiments by using a small sample size (*n* = 5 per group). Moreover, many animals are unable to elicit fear or nervousness because of their distinct biological or mental structures.

## 5 Conclusion

Our study demonstrates the sedative effectiveness of AA in a dose‐dependent manner by considerably reducing sleep latency and prolonging sleep duration in a mouse model of TS‐induced sleep. Notably, AA exhibits a synergistic interaction with DZP, further enhancing its sleep‐inducing effects. The in silico analysis confirms the drug‐likeness of AA, with favorable pharmacokinetic properties supporting its potential for further development. Molecular docking studies reveal a strong binding affinity of AA for GABA_A_ receptors, with a binding value of –7.9 kcal/mol, comparable to that of the reference drug DZP –8.4 kcal/mol. The combined administration of AA and DZP leads to a more pronounced sedative effect than either compound alone, reinforcing the therapeutic relevance of their interaction. While these findings suggest that AA is a viable option for sedative drug development, further studies are required to elucidate its precise mechanisms of action, optimize dosing strategies, and establish a comprehensive safety and efficacy profile before advancing toward clinical applications.

## Author Contributions

**Meher Afroz**: conceptualization (equal); formal analysis (equal); writing – original draft (equal); writing – review and editing (equal). **Rubel Hasan**: conceptualization (equal); formal analysis (equal); writing – original draft (equal); writing – review and editing (equal). **Mst Muslima Khatun**: data curation (supporting); resources (supporting). **Mokammel Hossain Tito**: methodology (supporting). **Md Shadin**: software (supporting). **Ranjit Chakma**: software (supporting). **Mohammed Asiri**: resources (supporting). **Faisal H. Altemani**: project administration (supporting). **Abdullah H. Altemani**: resources (supporting). **Md Shimul Bhuia**: validation (supporting); visualization (supporting). **Muhammad** **Torequl Islam**: supervision (supporting).

## Funding

This work was supported by Deanship of Scientific Research at King Khalid University (R.G.P.02/709/46).

## Conflicts of Interest

The authors declare no conflicts of interests.

## Data Availability

The data that support the findings of this study are available from the corresponding author upon reasonable request.

## References

[1] M. G. de Lima Silva , L. Y. S. da Silva , R. T. Pessoa , et al., “Antiedematogenic and Analgesic Activities of Abietic Acid in Mice,” Chemistry & Biodiversity 20 (2023): e202300906. 37795905 10.1002/cbdv.202300906

[2] K.‐H. Hwang , J.‐Y. Ahn , S. Kim , J.‐H. Park , and T.‐Y. Ha , “Abietic Acid Has an Anti‐Obesity Effect in Mice Fed a High‐Fat Diet,” Journal of Medicinal Food 14 (2011): 1052–1056. 21812648 10.1089/jmf.2010.1471

[3] B. Ahmad , C. Tian , J.‐X. Tang , J. S. Dumbuya , W. Li , and J. Lu , “Anticancer Activities of Natural Abietic Acid,” Frontiers in Pharmacology 15 (2024): 1392203, 10.3389/fphar.2024.1392203. 38633616 PMC11021724

[4] N. Shahzad , I. A. A. Ibrahim , A. R. Alzahrani , et al., “A Comprehensive Review on Phytochemicals as Potential Therapeutic Agents for Stress‐Induced Gastric Ulcer,” Journal of Umm Al‐Qura University for Applied Sciences 10 (2024): 793–808.

[5] E. J. Tettevi , M. Maina , D. L. Simpong , M. Y. Osei‐Atweneboana , and A. Ocloo , “A Review of African Medicinal Plants and Functional Foods for the Management of Alzheimer's Disease‐Related Phenotypes, Treatment of HSV‐1 Infection and/or Improvement of Gut Microbiota,” Journal of Evidence‐Based Complementary & Alternative Medicine 27 (2022): 2515690X221114657. 10.1177/2515690X221114657 PMC9310297 35866220

[6] R. Hasan , M. S. Bhuia , R. Chowdhury , et al., “Abietic Acid Antagonizes the Anti‐Inflammatory Effects of Celecoxib and Ketoprofen: Preclinical Assessment and Molecular Dynamic Simulations,” Computers in Biology and Medicine 183 (2024): 109298. 39454522 10.1016/j.compbiomed.2024.109298

[7] S. Kang , J. Zhang , and Y. Yuan , “Abietic Acid Attenuates IL‐1β‐Induced Inflammation in Human Osteoarthritis Chondrocytes,” International Immunopharmacology 64 (2018): 110–115. 30172103 10.1016/j.intimp.2018.07.014

[8] C. M. Morin , C. L. Drake , A. G. Harvey , et al., “Insomnia Disorder,” Nature Reviews. Disease Primers 1 (2015): 15026. 10.1038/nrdp.2015.26 27189779

[9] C. M. Morin and R. Benca , “Chronic Insomnia,” The Lancet 379 (2012): 1129–1141. 10.1016/S0140-6736(11)60750-2 22265700

[10] G. Medic , M. Wille , and M. E. Hemels , “Short‐ and Long‐Term Health Consequences of Sleep Disruption,” Nature and Science of Sleep 9 (2017): 151–161. 10.2147/NSS.S134864 PMC5449130 28579842

[11] K. C. Byars , K. Yolton , J. Rausch , B. Lanphear , and D. W. Beebe , “Prevalence, Patterns, and Persistence of Sleep Problems in the First 3 Years of Life,” Peds 129 (2012): e276–e284. 10.1542/peds.2011-0372 PMC3357046 22218837

[12] S. A. Mukty , R. Hasan , Md. S. Bhuia , et al., “Assessment of Sedative Activity of Fraxin: In Vivo Approach along with Receptor Binding Affinity and Molecular Interaction with GABAergic System,” Drug Development Research 85 (2024): e22250. 39154218 10.1002/ddr.22250

[13] A. Testa , R. Giannuzzi , F. Sollazzo , L. Petrongolo , L. Bernardini , and S. Daini , “Psychiatric Emergencies (part II): Psychiatric Disorders Coexisting with Organic Diseases,” European Review for Medical and Pharmacological Sciences 17 (2013): 65. 23436669

[14] P. Wańkowicz , A. Szylińska , and I. Rotter , “The Impact of the COVID‐19 Pandemic on Psychological Health and Insomnia among People with Chronic Diseases,” Journal of Clinical Medicine 10 (2021): 1206. 33799371 10.3390/jcm10061206 PMC7998391

[15] J. Ferdous , Md S. Bhuia , R. Chowdhury , et al., “Modulatory Sedative Activity of Abrine on Diazepam in Thiopental Sodium Mediated Sleeping Mice: An In Vivo Approach with Receptor Binding Affinity of GABAergic Transmission,” ChemistrySelect 9 (2024): e202403725.

[16] J. Brohan and B. G. Goudra , “The Role of GABA Receptor Agonists in Anesthesia and Sedation,” CNS Drugs 31 (2017): 845–856. 29039138 10.1007/s40263-017-0463-7

[17] M. Terunuma , “Diversity of Structure and Function of GABAB Receptors: A Complexity of GABAB‐Mediated Signaling,” Proceedings of the Japan Academy, Series B 94 (2018): 390–411. 10.2183/pjab.94.026 PMC6374141 30541966

[18] X. Qian , X. Zhao , L. Yu , et al., “Current Status of GABA Receptor Subtypes in Analgesia,” Biomedicine & Pharmacotherapy 168 (2023): 115800. 37935070 10.1016/j.biopha.2023.115800

[19] V. M. Patil and S. P. Gupta , “Studies on Chloride Channels and Their Modulators,” Current Topics in Medicinal Chemistry 16 (2016): 1862–1876. 26667116 10.2174/1568026616666151215104302

[20] A. C. Dolphin , “Functions of Presynaptic Voltage‐Gated Calcium Channels,” Function 2 (2021): zqaa027. 33313507 10.1093/function/zqaa027 PMC7709543

[21] A. Ghit , D. Assal , A. S. Al‐Shami , and D. E. E. Hussein , “GABAA Receptors: Structure, Function, Pharmacology, and Related Disorders,” Journal, Genetic Engineering & Biotechnology 19 (2021): 123. 10.1186/s43141-021-00224-0 PMC8380214 34417930

[22] Md T. Islam , Md S. Bhuia , S. Sheikh , et al., “Sedative Effects of Daidzin, Possibly Through the GABAA Receptor Interaction Pathway: In Vivo Approach with Molecular Dynamic Simulations,” Journal of Molecular Neuroscience 74 (2024): 83. 39230641 10.1007/s12031-024-02261-z

[23] S. Saini , S. Tahlan , and N. Minocha , “Current Therapeutic Strategies for the Management of Benzodiazepine (BZD) Withdrawal Syndrome: A Review,” Current topics in medicinal chemistry 24 (2024): 1529–1541. 38738726 10.2174/0115680266296096240408032738

[24] J. M. Kitzen , in: Naturally Occurring Benzodiazepines, Endozepines, and Their Receptors (CRC Press, 2021).

[25] E. J. Khantzian and G. J. McKenna , “Acute Toxic and Withdrawal Reactions Associated with Drug Use and Abuse,” Annals of Internal Medicine 90 (1979): 361–372. 34343 10.7326/0003-4819-90-3-361

[26] M. Mathur and M. T. Malik , Sedation and Analgesia for the Pediatric Intensivist: A Clinical Guide eds., P. P. Kamat and J. W. Berkenbosch (Springer Int. Publ, 2021), 401–410.

[27] R. Hasan , A. Alshammari , N. A. Albekairi , et al., “Antiemetic Activity of Abietic Acid Possibly through the 5HT3 and Muscarinic Receptors Interaction Pathways,” Scientific Reports 14 (2024): 6642. 38503897 10.1038/s41598-024-57173-0 PMC10951218

[28] L. D. Stein , “Towards a Cyberinfrastructure for the Biological Sciences: Progress, Visions and Challenges,” Nature Reviews Genetics 9 (2008): 678–688. 10.1038/nrg2414 18714290

[29] M. S. Bhuia , T. Islam , M. Rokonuzzman , et al., “Modulatory Effects of Phytol on the Antiemetic Property of Domperidone, Possibly through the D2 Receptor Interaction Pathway: In Vivo and in Silico Studies, 3 Biotech 13 (2023): 116. 10.1007/s13205-023-03520-3 PMC10008523 36919029

[30] M. Afroz , M. S. Bhuia , M. A. Rahman , et al., “Anti‐Diarrheal Effect of Piperine Possibly through the Interaction with Inflammation Inducing Enzymes: *In Vivo* and *in Silico* Studies,” European Journal of Pharmacology 965 (2024): 176289. 38158111 10.1016/j.ejphar.2023.176289

[31] R. Chowdhury , M. S. Bhuia , A. I. Rakib , et al., “Assessment of Quercetin Antiemetic Properties: In Vivo and In Silico Investigations on Receptor Binding Affinity and Synergistic Effects,” Plants 12 (2023): 4189. 38140516 10.3390/plants12244189 PMC10747098

[32] M. H. Bappi , M. N. Mia , S. A. Ansari , et al., “Quercetin Increases the Antidepressant‐Like Effects of Sclareol and Antagonizes Diazepam in Thiopental Sodium‐Induced Sleeping Mice: A Possible GABAergic Transmission Intervention,” Phytotherapy Research 38 (2024): 2198–2214. 38414297 10.1002/ptr.8139

[33] F. Stanzione , I. Giangreco , and C. J., Cole in Progress in Medicinal Chemistry eds., D. R. Witty and B. Cox (Elsevier, 2021). 273–343. 10.1016/bs.pmch.2021.01.004 34147204

[34] R. Hasan , Md S. Bhuia , R. Chowdhury , et al., “Piperine Exerts Anti‐Inflammatory Effects and Antagonises the Properties of Celecoxib and Ketoprofen: In vivo and Molecular Docking Studies,” Natural Product Research 0 (2024): 1–16. 10.1080/14786419.2024.2413039 39390887

[35] H. Li , H. Su , A. Komori , et al., “Supercomputing Multi‐Ligand Modeling, Simulation, Wavelet Analysis and Surface Plasmon Resonance to Develop Novel Combination Drugs: A Case Study of Arbidol and Baicalein Against Main Protease of SARS‐CoV‐2,” Pharmaceuticals 18 (2025): 1054. 40732341 10.3390/ph18071054 PMC12300969

[36] M. Rizk , L. Zou , R. Savic , and K. Dooley , “Importance of Drug Pharmacokinetics at the Site of Action,” Clinical and Translational Science 10 (2017): 133–142. 28160433 10.1111/cts.12448 PMC5421734

[37] M. Shah , M. Patel , M. Shah , M. Patel , and M. Prajapati , “Computational Transformation in Drug Discovery: A Comprehensive Study on Molecular Docking and Quantitative Structure Activity Relationship (QSAR,” Intelligent Pharmacy 2 (2024): 589–595.

[38] A. M. B. Amorim , L. F. Piochi , A. T. Gaspar , A. J. Preto , N. Rosário‐Ferreira , and I. S. Moreira , “Advancing Drug Safety in Drug Development: Bridging Computational Predictions for Enhanced Toxicity Prediction,” Chemical Research in Toxicology 37 (2024): 827–849. 38758610 10.1021/acs.chemrestox.3c00352 PMC11187637

[39] A. D. Vecchia , A. Arone , A. Piccinni , F. Mucci , and D. Marazziti , “GABA System in Depression: Impact on Pathophysiology and Psychopharmacology,” Current Medicinal Chemistry 29 (2022): 5710–5730. 34781862 10.2174/0929867328666211115124149

[40] M. M. Khatun , M. S. Bhuia , M. Rahaman , H. Kamli , S. A. Ansari , and M. T. Islam , “Modulatory Sedative Activity of Helicide on Diazepam in Thiopental Sodium Mediated Sleeping Mice: An In Vivo Approach With Molecular Docking,” Revista Brasileira De Farmacognosia 35 (2025): 412–421.

[41] M. S. Bhuia , H. Kamli , T. Islam , et al., “Antiemetic Activity of *Trans*‐Ferulic Acid Possibly through Muscarinic Receptors Interaction Pathway: In vivo and *in Silico* Study,” Results in Chemistry 6 (2023): 101014.

[42] H. G. Bagewadi , “An Experimental Study to Evaluate the Effect of Memantine in Animal Models of Anxiety in Swiss Albino Mice,” Journal of Clinical and Diagnostic Research 9 (2015): FF01–FF5, 10.7860/JCDR/2015/13233.6287. PMC4576556 26435964

[43] J. J. Kim and R. E. Hibbs , “Direct Structural Insights into GABAA Receptor Pharmacology,” Trends in Biochemical Sciences 46 (2021): 502–517. 33674151 10.1016/j.tibs.2021.01.011 PMC8122054

[44] I. Tobler , C. Kopp , T. Deboer , and U. Rudolph , “Diazepam‐Induced Changes in Sleep: Role of the *α*1 GABAA Receptor Subtype,” Proceedings of the National Academy of Sciences U.S.A 98 (2001): 6464–6469. 10.1073/pnas.111055398 PMC33491 11353839

[45] N. Li , J. Liu , M. Wang , et al., “Sedative and Hypnotic Effects of Schisandrin B through Increasing GABA/Glu Ratio and Upregulating the Expression of GABAA in Mice and Rats,” Biomedicine & Pharmacotherapy 103 (2018): 509–516. 29677536 10.1016/j.biopha.2018.04.017

[46] M. S. A. Hasan , M. S. Bhuia , R. Chowdhury , et al., “Tangeretin Enhances Sedative Activity of Diazepam in Swiss Mice through GABAA Receptor Interaction: In vivo and in Silico Approaches,” Neuroscience 572 (2025): 1–10. 40049390 10.1016/j.neuroscience.2025.03.004

[47] A. B. Philip , J. Brohan , and B. Goudra , “The Role of GABA Receptors in Anesthesia and Sedation: An Updated Review,” CNS Drugs 39 (2025): 39–54. 39465449 10.1007/s40263-024-01128-6 PMC11695389

[48] M. Pádua‐Reis , D. A. Nôga , A. B. L. Tort , and M. Blunder , “Diazepam Causes Sedative rather than Anxiolytic Effects in C57BL/6J Mice,” Scientific Reports 11 (2021): 9335. 33927265 10.1038/s41598-021-88599-5 PMC8085115

[49] R. Pezzani , B. Salehi , S. Vitalini , et al., “Synergistic Effects of Plant Derivatives and Conventional Chemotherapeutic Agents: An Update on the Cancer Perspective,” Medicina 55 (2019): 110. 30999703 10.3390/medicina55040110 PMC6524059

[50] P. C. Agu , C. A. Afiukwa , O. U. Orji , et al., “Molecular Docking as a Tool for the Discovery of Molecular Targets of Nutraceuticals in Diseases Management,” Scientific Reports 13 (2023): 13398. 37592012 10.1038/s41598-023-40160-2 PMC10435576

[51] M. R. Duff and E. E. Howell , “Thermodynamics and Solvent Linkage of Macromolecule‐Ligand Interactions,” Methods 76 (2015): 51–60. 25462561 10.1016/j.ymeth.2014.11.009 PMC4380819

[52] D. Chen , N. Oezguen , P. Urvil , C. Ferguson , S. M. Dann , and T. C. Savidge , “Regulation of Protein‐Ligand Binding Affinity by Hydrogen Bond Pairing,” Science Advances 2 (2016): e1501240. 27051863 10.1126/sciadv.1501240 PMC4820369

[53] M. Thakur , S. Kumari , S. Kumar , and M. Kumari , “Integrated Quantum Chemical Calculations, Predictive Toxicity Assessment, Absorption, Distribution, Metabolism, Excretion and Toxicity Profiling and Molecular Docking Analysis to Unveil the Therapeutic Potential of Non‐Oxovanadium(IV) and Organotin(IV) Complexes Targeting Breast Cancer Cells,” International Journal of Quantum Chemistry 124 (2024): e27438.

[54] I. Yusof and M. D. Segall , “Considering the Impact Drug‐Like Properties Have on the Chance of Success,” Drug Discovery Today 18 (2013): 659–666. 23458995 10.1016/j.drudis.2013.02.008

[55] C. Agoni , F. A. Olotu , P. Ramharack , and M. E. Soliman , “Druggability and Drug‐Likeness Concepts in Drug Design: Are Biomodelling and Predictive Tools Having Their Say?,” Journal of Molecular Modeling 26 (2020): 120. 32382800 10.1007/s00894-020-04385-6

[56] R. C. Mohs and N. H. Greig , “Drug Discovery and Development: Role of Basic Biological Research,” Alzheimer's & Dementia (Translational Research & Clinical Interventions) 3 (2017): 651–657. 10.1016/j.trci.2017.10.005 PMC5725284 29255791

[57] J. N. Chu and G. Traverso , “Foundations of Gastrointestinal‐Based Drug Delivery and Future Developments,” Nature Reviews Gastroenterology & Hepatology 19 (2022): 219–238. 34785786 10.1038/s41575-021-00539-w PMC12053541

[58] Z.‐Y. Wu , J. Pan , Y. Yuan , A.‐L. Hui , Y. Yang , and A. Zhou , “Comparison of Prediction Models for Blood Brain Barrier Permeability and Analysis of the Molecular Descriptors,” Die Pharmazie 67 (2012): 628–634. 22888521

[59] S. Shityakov , W. Neuhaus , T. Dandekar , and C. Förster , “Analysing Molecular Polar Surface Descriptors to Predict Blood‐Brain Barrier Permeation,” International Journal of Computational Biology and Drug Design 6 (2013): 146. 23428480 10.1504/IJCBDD.2013.052195

[60] L. M. Schuijt , F.‐J. Peng , S. J. P. van den Berg , M. M. L. Dingemans , and P. J. Van den Brink , “Eco)toxicological Tests for Assessing Impacts of Chemical Stress to Aquatic Ecosystems: Facts, Challenges, and Future,” The Science of the Total Environment 795 (2021): 148776. 34328937 10.1016/j.scitotenv.2021.148776

[61] P. A. Thompson , M. Khatami , C. J. Baglole , et al., “Environmental Immune Disruptors, Inflammation and Cancer Risk,” Carcinogenesis 36 (2015): S232–S253. 26106141 10.1093/carcin/bgv038 PMC4492068

[62] A. Sabarwal , K. Kumar , and R. P. Singh , “Hazardous Effects of Chemical Pesticides on Human Health–Cancer and Other Associated Disorders,” Environmental Toxicology and Pharmacology 63 (2018): 103–114. 30199797 10.1016/j.etap.2018.08.018

[63] S. Alhaji Saganuwan , “Toxicity Studies of Drugs and Chemicals in Animals: An Overview,” Bulgarian Journal of Veterinary Medicine 20 (2017): 291–318.

[64] V. M. Ahur , S. M. Anika , and P. A. Onyeyili , “Age‐Sex Dimorphisms in the Estimation of Median Lethal Dose (LD50) of Lead Diacetate in Rabbits using up‐and‐Down Procedure (Arithmetic Method,” Sokoto Journal of Veterinary Sciences 16 (2018): 64–72.

[65] A. L. Parra , R. S. Yhebra , I. G. Sardiñas , and L. I. Buela , “Comparative Study of the Assay of *Artemia Salina L.* and the Estimate of the Medium Lethal Dose (LD50 Value) in Mice, to Determine Oral Acute Toxicity of Plant Extracts,” Phytomedicine 8 (2001): 395–400. 11695884 10.1078/0944-7113-00044

